# Quality of Mobile Apps for Care Partners of People With Alzheimer Disease and Related Dementias: Mobile App Rating Scale Evaluation

**DOI:** 10.2196/33863

**Published:** 2022-03-29

**Authors:** Nicole E Werner, Janetta C Brown, Priya Loganathar, Richard J Holden

**Affiliations:** 1 Department of Industrial and Systems Engineering University of Wisconsin-Madison Madison, WI United States; 2 Indiana University School of Medicine Indianapolis, IN United States; 3 Department of Health & Wellness Design School of Public Health-Bloomington Indiana University Bloomington, IN United States

**Keywords:** Alzheimer disease and related dementias, mobile app, mHealth, caregivers, dementia caregiving, eHealth, telehealth, mobile phone

## Abstract

**Background:**

Over 11 million care partners in the United States who provide care to people living with Alzheimer disease and related dementias (ADRD) cite persistent and pervasive unmet needs related to their caregiving role. The proliferation of mobile apps for care partners has the potential to meet care partners’ needs, but the quality of apps is unknown.

**Objective:**

This study aims to evaluate the quality of publicly available apps for care partners of people living with ADRD and identify design features of low- and high-quality apps to guide future research and user-centered app development.

**Methods:**

We searched the US Apple App and Google Play stores with the criteria that included apps needed to be available in the US Google Play or Apple App stores, accessible to users out of the box, and primarily intended for use by an informal (family or friend) care partner of a person living with ADRD. We classified and tabulated app functionalities. The included apps were then evaluated using the Mobile App Rating Scale (MARS) using 23 items across 5 dimensions: engagement, functionality, aesthetics, information, and subjective quality. We computed descriptive statistics for each rating. To identify recommendations for future research and app development, we categorized rater comments on score-driving factors for each MARS rating item and what the app could have done to improve the item score.

**Results:**

We evaluated 17 apps. We found that, on average, apps are of minimally acceptable quality. Functionalities supported by apps included education (12/17, 71%), interactive training (3/17, 18%), documentation (3/17, 18%), tracking symptoms (2/17, 12%), care partner community (3/17, 18%), interaction with clinical experts (1/17, 6%), care coordination (2/17, 12%), and activities for the person living with ADRD (2/17, 12%). Of the 17 apps, 8 (47%) had only 1 feature, 6 (35%) had 2 features, and 3 (18%) had 3 features. The MARS quality mean score across apps was 3.08 (SD 0.83) on the 5-point rating scale (1=inadequate to 5=excellent), with apps scoring highest on average on functionality (mean 3.37, SD 0.99) and aesthetics (mean 3.24, SD 0.92) and lowest on average on information (mean 2.95, SD 0.95) and engagement (mean 2.76, SD 0.89). The MARS subjective quality mean score across apps was 2.26 (SD 1.02).

**Conclusions:**

We identified apps whose mean scores were more than 1 point below minimally acceptable quality, whereas some were more than 1 point above. Many apps had broken features and were rated as below acceptable for engagement and information. Minimally acceptable quality is likely to be insufficient to meet care partner needs. Future research should establish minimum quality standards across dimensions for care partner mobile apps. Design features of high-quality apps identified in this study can provide the foundation for benchmarking these standards.

## Introduction

### Background

Over 11 million care partners in the United States who provide care to people living with Alzheimer disease and related dementias (ADRD) are often untrained, underresourced, and unsupported to manage the cognitive, behavioral, and physical changes that characterize ADRD progression [1-3]. Therefore, care partners cite persistent and pervasive unmet needs related to all aspects of their caregiving role, including support for daily care, managing behavioral symptoms of dementia, self-care, resources and support services, health information management, care coordination and communication, and financial and legal planning [4-6]. The ability to address the unmet needs of care partners is a critical health challenge, as these unmet needs are associated with suboptimal psychological and physical outcomes for the care partner and the person living with ADRD [7-10].

National experts call for technologies to be powerful and novel interventions to support care partners [11]. For example, experts from the 2015 Alzheimer Disease Research Summit recommended to “develop new technologies that enhance the delivery of clinical care, care partner support, and in-home monitoring” and “test the use of technology to overcome the workforce limitations in the care of older adults with dementia as well as providing care partner support and education” [11]. The 2018 Research Summit called for “innovative digital data collection platforms” and “pervasive computing assessment methods” [12].

Mobile apps can answer these calls by enabling unique data capture and visualization, multichannel communication, and integration of powerful decision support on increasingly ubiquitous and scalable devices (eg, smartphones). Advancing technological capabilities also increase the potential of mobile apps to provide much-needed individualized, just-in-time support that can adapt to changing needs across the course of the disease [13]. Reviews of mobile apps for care partners report that they are a feasible and acceptable intervention [14] and can reduce ADRD care partner stress and burden [15].

The mere availability of apps is not sufficient to improve health outcomes; these apps must be designed to support and accommodate user needs and abilities, a process called user-centered design (UCD). More formally, UCD is:

an approach to interactive systems development that aims to make systems usable and useful by focusing on the users, their needs and requirements, and by applying human factors/ergonomics, usability knowledge, and techniques. This approach enhances effectiveness and efficiency, improves human well-being, user satisfaction, accessibility and sustainability, and counteracts possible adverse effects of use on human health, safety and performance.

16

UCD provides a scientifically sound, practice-based mechanism for developing mobile apps for care partners of people living with ADRD that are highly feasible and more likely to improve care partner outcomes [17]. Conversely, if apps are not designed using UCD, they are more likely to be of low quality, cause more harm than good, incur avoidable waste of financial and human resources, not provide the needed support, and compound the existing burden on care partners [13,16,18-21].

Despite the potential of mobile apps to meet care partners’ needs and improve outcomes using UCD and other industry-standard design practices, the actual quality of mobile apps for care partners—that is, how usable, engaging, valid, acceptable, accessible, aesthetically pleasing, and useful they are to the user—is currently unknown. A recent study by Choi et al [22] used the Mobile App Rating Scale (MARS) to assess app quality across ADRD-related apps focused on self-care management for people living with ADRD. They found that, on average, the evaluated apps met the MARS criteria for minimally acceptable quality, quality scores were higher for those developed by health care–related versus non–health care–related developers, and apps scored lower on average regarding how engaging they were to the user [22]. Although this study included some apps with care partners as the intended primary user, the inclusion and exclusion criteria focused on the person living with ADRD, which limited the inclusion of apps targeted at care partners as the intended end user.

It is critical to evaluate the quality of mobile apps for ADRD care partners for several reasons [17]. First, quality assessment ensures that mobile apps produce benefits and do not have unintended health consequences for care partners or persons living with ADRD; for example, they do not increase care partner stress and burden. Second, quality evaluation can provide insights into whether mobile apps will be used and whether use will withstand the test of time; that is, they will not be abandoned. Third, quality evaluation is important to ensure that research-based mobile apps are sustainable outside academic research settings, meaning they can achieve commercial success among competitors. Fourth, quality evaluation can safeguard against commercial products that may not deliver on their advertised potential.

### Objectives

Thus, the aim of this study is to (1) evaluate the quality of publicly available apps for care partners of people living with ADRD and (2) identify the design features of low- and high-quality apps to guide future research and user-centered app development.

## Methods

### Design

We conducted a multirater evaluation of the quality of mobile apps for caregivers of people living with ADRD available on the US market by applying the MARS [23]. The MARS was created to be an easy-to-use and objective tool for researchers and developers to evaluate the quality of mobile apps across multiple dimensions. We chose to use the MARS because it is a validated rating scale for mobile app quality, includes a multicomponent evaluation of quality, has clear instructions and a uniform scale, and has been used successfully across multiple health domains, including pain management and ADRD [22,24,25].

### Data Collection

#### App Identification and Selection

We searched the US Apple App and Google Play stores in March 2021 using multiple variations of the terms “caregiver,” “carer,” “care,” “caretaker,” “dementia,” and “Alzheimer disease.” To be included in the analysis, an app needed to be (1) available in US Google Play or Apple App stores; (2) directly accessible to users out of the box (ie, without a separate agreement with an insurer, health care delivery organization, and enrolling in a clinical trial); and (3) primarily intended for use by an informal (family or friend) care partner or care partners of a person with dementia of any severity, stage, or etiology. Four members of the research team independently searched both app stores to identify eligible apps based on the app name and brief description and identified 50 unique apps. Next, 3 members of the research team applied the inclusion criteria to the compiled list of apps by reviewing the full app description and downloading and exploring the app components. One research team member served as the arbiter by reviewing each app for inclusion and documenting the reason for inclusion or exclusion. The arbiter presented their inclusion decisions to the full research team for a consensus. Primary reasons for app exclusion were not having the caregiver as the primary user (eg, apps for the person living with ADRD), needing to sign up for a clinical trial or be part of a specific health system to access the app, and not being specific to ADRD care (eg, targeted for caregivers of people with any condition). We identified 17 unique apps that met our inclusion criteria, 8 (47%) of which were available in both the iOS and Android versions. For apps that were available on both iOS and Android, we randomly selected whether we would evaluate the iOS or Android version. An expert rater also reviewed the version that was not selected to assess quality differences, and no quality differences were identified between platforms for any of the apps.

#### App Classification

For each included app, we captured descriptive and technical information, such as name, ratings, version history, language, and functionality. We classified the app’s purpose and functionality based on the app store description and available functionality within the app.

#### MARS Evaluation

The MARS includes 23 items across 5 dimensions: engagement, functionality, aesthetics, information, and subjective quality [23]. Each item was scored on a 5-point scale, from inadequate (score=1) to excellent (score=5) or not applicable.

Our MARS evaluation team included 7 research team members: 3 experts in UCD and ADRD caregiving and 4 trainees in these areas. The MARS training process began with the full team independently reviewing the published MARS guide, including instructions, definitions, and rating scales. Next, we conducted 3 team-based training sessions to improve consensus on the MARS ratings. During the training sessions, we evaluated each app as a team, item by item, with a discussion of each item rating to build consensus on how to interpret the items and the criteria for each score within an item. During the team rating sessions, we discussed score anchors and annotated the MARS rating sheet based on consensus anchors. Between team training sessions, team members practiced applying the ratings discussed in the previous session and created additional annotations based on the team consensus discussion, which were then shared with the full team at the subsequent meeting.

Next, each app was rated using the MARS by at least 2 independent raters. To apply the MARS, each trained rater downloaded the app to a testing phone, paid fees, and tested the app to ensure that all components of the app were used. The rater then completed the 23 MARS rating items in order, app by app. In addition to the required MARS rating procedures, raters also documented for each item the score-driving factors for that item and what the app could have done to improve the score. This was done to support our aim of guiding future research and app development.

Two members of the research team reviewed all the scores and identified the items for which the original 2 raters had disagreements in their scores. For items with a disagreement score, an expert rater (JCB, RJH, or NEW) was used as the tiebreaker. The goal of the expert rater as a tiebreaker was to determine which of the scores they agreed with are based on the MARS training and their expertise in evaluating health information technologies. However, if the expert rater disagreed with both scores, the tie-breaking score could be different from the original 2 raters with clear justification. Expert raters were senior members of the research team with doctoral training in UCD, a combined 6 years designing and evaluating dementia caregiving technologies, and a combined 13 years evaluating health information technologies. We calculated the percentage agreement for each rating dyad and the overall agreement rate.

### Data Analysis

The ratings were entered into a cloud-based Microsoft Excel spreadsheet, and descriptive statistics were computed for each rating. First, we computed the mean score for each of the quality dimensions (engagement, functionality, aesthetics, information, and subjective quality) for each individual app as the sum of the item scores in each dimension divided by the items in the dimension. Next, we calculated the app quality mean score for each app as the sum of the dimension mean scores divided by the number of dimensions. We calculated the total mean score for each dimension across all apps as the sum of each app’s dimension mean score divided by the total number of apps. We computed the overall app subjective mean score as the sum of the mean scores divided by the total number of apps.

To identify recommendations for future research and app development, 2 expert members of the research team categorized rater comments on the score-driving factors for each item and what the app could have done to improve the score for that item. They then met to discuss the categories and reach a consensus.

### Ethics Approval

This study did not involve human subjects.

## Results

### App Classification

We evaluated 17 apps (n=7, 41%, iOS only; n=2, 12%, Android only; and n=8, 47%, both iOS and Android). Before the expert-based score reconciliation process, across 6 rating dyads, the raters provided the exact same rating on a 1 to 5 scale in 43% of ratings; rater dyads agreed within 1 point in 83% of cases (detailed agreement and disagreement rates of each rating dyad are given in Multimedia Appendix 1). All apps except one were available at no cost for the most basic version, and no apps required an additional cost to upgrade to advanced features or additional content. Apps had affiliations with commercial companies 47% (8/17), universities 24% (4/17), health systems 18% (3/17), governments 12% (2/17), and nongovernmental organizations 6% (1/17). Of the 17 apps evaluated, 14 (82%) were available in English only; 1 (6%) was available in English, Korean, and Spanish; 1 (6%) was available in English and Japanese; and 1 (6%) was available in English and Portuguese. Full descriptions and technical details of the apps are provided in Table 1.

We identified 8 general feature categories supported by the apps (Table 2). These categories included the ability of the app to provide the following: (1) education—the provision of relevant, appropriate content that increases care partner knowledge and self-efficacy to perform their role and make informed decisions (12/17, 71%); (2) interactive training—reciprocal exchange of information for care partner development and learning (3/17, 18%); (3) documentation—storage or recording of information for later retrieval (3/17, 18%); (4) tracking of symptoms (2/17, 12%); (5) care partner community—a platform or feature created for the exchange of social support among care partners (3/17, 18%); (6) interaction with clinical experts (1/17, 6%); (7) care coordination—the organization and distribution of patient care activities among all involved participants (2/17, 12%); and (8) activities for the person living with ADRD (2/17, 12%). Of the 17 apps, 8 (47%) had only 1 feature, 6 (35%) had 2 features, and 3 (18%) had 3 features. Most apps (12/17, 71%) provided information, and for 29% (5/17) of apps, providing information was the only feature. Of the 17 apps, some features such as interaction with clinicians or tracking symptoms were offered by only 1 (6%) or 2 (12%) apps.

**Table 1:** Apps evaluated in the study and their descriptive and technical details.

App name	Platform	Category	Developer	Year of last update	Country	Language	Purpose	Affiliations
Accessible Alzheimer's and Dementia Care	iOS	Health and fitness	ACHGLOBAL, Inc	N/A^a^	United States	English	Provide valuable information	Commercial
Alzheimer's and Dementia Care	Android	Health and fitness	Accessible home health care	2019	United States	English	Provide information	Commercial
Alzheimer’s Daily Companion	iOS^b^ Android	Lifestyle	Home Instead Senior Care	2013	United States	English	Build care partner confidence	Commercial
Alzheimer’s Manager	iOS	Health and fitness	Point of Care LLC	2021	United States	English	Track and monitor	Commercial
Care4Dementia	iOS	Medical	Univ of New South Wales	N/A	Australia	English	Provide information	University
Clear Dementia Care	iOS Android	Medical	Northern Health and Social Care Trust	2021	United States	English	Provide information and support	Government Health System
CogniCare	iOS^b^ Android	Health and fitness	CongniHealth Ltd	2021	United Kingdom	English, Japanese	Improve quality of life	Commercial University
Dementia Advisor	iOS^b^ Android	Health and fitness	Sinai Health System	2019	Canada	English	Improve communication	Government Health System
Dementia Caregiver Solutions	iOS^c^	Health	Lorenzo Gentile	2016	Canada	English	Provide expert advice	Commercial
Dementia Guide Expert	iOS^b^ Android	Education	University of Illinois	2020	United States	English, Korean, Spanish	Educate and empower	University
Dementia Stages Ability Model	iOS^b^ Android	Education	Positive Approach, LLC	2020	United States	English	Help learn characteristics and care of GEMS stages	Commercial
Dementia Talk	iOS	Health and fitness	Sinai Health System- Reitman Centre	2019	United States	English	Track and monitor	Health System
DementiAssist	Android	Medical	Baylor Scott and White Health	2015	United States	English	Provide insights	University
DemKonnect	iOS^b^ Android	Medical	Nightingales Medical Trust	2020	United Kingdom	English	Provide care partner connections	NGO^d^
Inspo-Alzheimer’s Caregiving	iOS^b^ Android	Social networking	Inspo Labs	2020	United States	English	Create safe supportive community	N/A
Remember Me-Caregiver	iOS	Productivity	Daniel Leal	2020	United States	English, Portuguese	Share care responsibility	N/A
Respite Mobile	iOS	Health and fitness	ADC initiatives LLC	2021	United States	English	Provide activities for people with ADRD^e^	Commercial

^a^N/A: not available.

^b^Indicates platform reviewed.

^c^Indicates cost of use.

^d^NGO: nongovernmental organization.

^e^ADRD: Alzheimer disease and related dementias.

**Table 2:** Feature categories of the evaluated apps (N=17).

Apps	Education (n=12, 71%)	Interactive training (n=3, 18%)	Documentation (n=3, 18%)	Tracking symptoms (n=2, 12%)	Care partner community (n=3, 18%)	Interaction with clinical expert (n=1, 6%)	Care coordination (n=2, 12%)	Activities for person with dementia (n=2, 12%)
Accessible Alzheimer's and Dementia Care	✓							
Alzheimer's and Dementia Care	✓				✓			
Alzheimer's Daily Companion	✓							
Alzheimer's Manager			✓	✓				
Care4Dementia	✓							
Clear Dementia Care	✓	✓	✓					
CogniCare	✓							✓
Dementia Advisor		✓						
Dementia Caregiver Solutions	✓							
Dementia Guide Expert	✓							
Dementia Stages Ability Model	✓	✓						
Dementia Talk			✓	✓			✓	
DementiAssist	✓							
DemKonnect	✓				✓	✓		
Inspo-Alzheimer’s Caregiving	✓				✓			
Remember Me-Caregiver							✓	
Respite Mobile								✓

### MARS Evaluation

The MARS app quality mean score across all apps was 3.08 (SD 0.83) on the 5-point rating scale (from 1=inadequate to 5=excellent), with apps scoring highest on average on functionality (mean 3.37, SD 0.99) and aesthetics (mean 3.24, SD 0.92) and lowest on average on information (mean 2.95, SD 0.95) and engagement (mean 2.76, SD 0.89; Table 3).

**Table 3:** Mean scores on the Mobile App Rating Scale (MARS) rating categories with category definitions and subjective evaluation data, including app store number of ratings, app store average ratings, and the MARS subjective quality score.

Apps	Quality mean score, mean	Engagement, mean	Functionality, mean	Aesthetics, mean	Information, mean	Subjective quality score, mean	App store number ratings	App store average rating (out of 5 stars)
Accessible Alzheimer’s and Dementia Care	1.26	1.20	1.50	1.00	1.33	1.00	1	5
Alzheimer’s and Dementia Care	2.29	2.00	2.00	2.67	2.50	1.00	NR^a^	NR
Alzheimer’s Daily Companion	2.50	1.60	3.25	2.67	2.50	1.25	16	4.6
Alzheimer’s Manager	2.98	2.60	3.50	3.00	2.83	2.67	1	3
Care4Dementia	3.20	2.20	3.75	3.00	3.83	2.50	1	5
Clear Dementia Care	3.85	3.80	4.25	3.33	4.00	3.25	1	5
CogniCare	3.74	3.80	4.00	4.00	3.17	2.50	NR	NR
Dementia Advisor	4.18	4.20	5.00	3.33	4.17	4.50	6	4.3
Dementia Caregiver Solutions	3.11	2.60	3.50	3.00	3.33	2.00	1	5
Dementia Guide Expert	2.66	2.40	2.75	2.33	3.17	3.25	1	5
Dementia Stages Ability Model	4.26	3.30	4.75	4.67	4.33	3.25	15	5
Dementia Talk	3.29	3.00	3.00	4.00	3.17	1.25	1	1
DementiAssist	2.93	2.80	3.25	3.33	2.33	2.00	7	4.1
DemKonnect-	2.71	3.00	2.00	3.67	2.17	1.75	NR	NR
Inspo-Alzheimer’s Caregiving	4.17	4.00	4.00	4.67	4.00	3.25	NR	NR
Remember Me-Caregiver	1.88	1.50	2.50	2.33	1.17	1.00	NR	NR
Respite Mobile	3.35	3.00	4.25	4.00	2.17	2.00	10	5
Overall, mean (SD)	3.08 (0.83)	2.76 (0.89)	3.37 (0.99)	3.24 (0.92)	2.95 (0.95)	2.26 (1.02)	N/A^b^	N/A

^a^NR: not rated.

^b^N/A: not applicable.

The MARS subjective quality mean score across all apps was 2.26 (SD 1.02), with mean scores ranging from 1 to 4.5. The mean score for the question, “Would you recommend the app to people who might benefit from it?” was 2.59 (SD 1.42).

The MARS app quality mean score of 2.94 (SD 0.93) for apps with a commercial affiliation was slightly below the minimally acceptable quality and slightly above the minimally acceptable quality 3.26 (SD 0.57) for apps with noncommercial affiliations (ie, universities, governments, health systems, and nongovernmental organizations). The MARS subjective quality mean score (SD) was below the minimally acceptable quality for apps with both commercial affiliation (mean 1.96, SD 0.83) and noncommercial affiliations (mean 2.64, SD 1.11).

Table 3 provides the mean scores on the MARS quality rating dimensions and subjective evaluation data, including the MARS subjective quality score, app store number of ratings, and app store average ratings for all evaluated apps.

### Score-Driving Factors

Table 4 lists the most frequently identified design qualities that led to low or high MARS scores for each MARS dimension. Among factors contributing to low scores, a common one was broken functionality, leading to crashes, error messages, and unresponsiveness, noted in 59% (10/17) of the apps. Among factors contributing to high scores, a common quality included aesthetics where adequate use of multimedia for content presentation, clear and consistent user interface layouts, and high-quality graphics were noted in 53% (9/17) of the apps.

**Table 4:** Mobile App Rating Scale (MARS) dimensions, categories within those dimensions, and examples of design features that were score drivers for low and high scores.

MARS dimension and categories		Examples of low-score drivers	Examples of high-score drivers
**Engagement**			
	Entertainment	Entertaining content such as games, chat, videos, and forums that do not function; extensive and overwhelming content; and very little content	Use of multimedia (eg, combination of text, video, audio, images, and animations)
	Interest	Text only with no images, large blocks of text, frequent system failure, constantly linking to outside website, and no ability to customize experience	Variety of content, features, and color throughout the app
	Customization	Limited, inoperable, or missing customization features	Variety of customization options (eg, privacy settings, preference selection, notifications, and favorites)
	Interactivity	Interactive content such chat, graphs, and forums does not function; must click what you need every time (the app does not retain information); and community forum but no active users	Feedback systems (eg, confirmations, error messages, and validations) and variety of data visualization with charts, in-app messaging, and features for community building
	Target group	Small font, no ability to zoom, provides only general information, and no privacy settings	Content relevance and usefulness of information
**Functionality**			
	Performance	Frequent error messages and crashes, frequently unresponsive or slow, and includes inactive hyperlinks	Responsiveness and efficient transitions throughout the app
	Ease of use	Takes a lot of time to figure out how to use, functions difficult to learn to use the complicated app architecture, and no instructions provided	Clarity and intuitiveness of app functions and learnability, operability, and app instructions
	Navigation	Menu options change within the app, clicking a link takes you to the incorrect function, consistently sending to an outside website with broken links, no back button provided, and the Menu options do not function	Logic, consistency, and visual cues matched users’ expectations; external sources within the app; and minimalist design
	Gestural design	Gestures differ from expectation in terms of phone gestures	Logical, consistent, anticipated gestures, links, and buttons
**Aesthetics**			
	Layout	Blocks of text and inconsistent layout across pages	Clear and simple user interface layout
	Graphics	Low quality (blurry) and no graphics	Quality, high-resolution graphics
	Visual appeal	No color, no graphics, no multimedia, and inconsistent text sizes and colors	Creative, impactful, and thoughtful use of color
**Information**			
	Accuracy of app description	Describes content that does not function or is not available	Features and functions aligned with the app description
	Goals	Goals not stated, goals not achievable because app functions are broken or unresponsive, and no ability to measure goal attainment	Goals stated explicitly with measurable or trackable metrics
	Quality of information	Sources not cited, sources cited are questionable, links provided to sources are broken, and information disorganized or difficult to locate	Information provided from trusted, cited sources; language used written with end users or target demographic in mind; and information relevant to users
	Quantity of information	Extensive and overwhelming amount of information and very little information	Sufficient and comprehensive range of information
	Visual information	No visual information available	Logical use of videos, multimedia, and helpful images to provide clarity
	Credibility	No sources cited and commercial entity selling other products	Created by a legitimate, verified entity, including hospital, center, government, university, or council
	Evidence base	Not tested for effectiveness in improving person living with ADRD^a^ or care partner outcomes	N/A^b^

^a^ADRD: Alzheimer disease and related dementias.

^b^N/A: not available.

## Discussion

### Principal Findings

The objectives of our study were to (1) evaluate the quality of publicly available apps for care partners of people living with ADRD and (2) identify the design features of low- and high-quality apps to guide future research and app development. Our findings show that across all apps, the average MARS quality rating was just above the minimally acceptable cut-off of 3.00 (mean 3.08, SD 0.83; range 1.26-4.26), and the average MARS subjective quality rating of all the apps was less than acceptable (mean 2.26, SD 1.02; range 1.00-4.50). We also identified apps whose individual mean scores were more than 1 point below the minimal acceptable quality, whereas some were more than 1 point above. Furthermore, most of the apps we assessed had broken features and were rated as below acceptable quality for the MARS dimensions of engagement and information quality.

Of the 17 mobile apps, our analysis identified 3 (18%) with a rating of good or higher quality (MARS quality mean score >4). Furthermore, Dementia Advisor scored greater than 4 (ie, indicating good quality) on both the MARS quality mean score and the subjective quality mean score. In contrast to most apps that focus on providing education through text and videos, Dementia Advisor provides interactive training on a wide variety of scenarios with feedback to improve learning. The app was simple and intuitive, without the need for instructions or significant time to learn to use the app features. All features of the app were functional, and the progress through the training scenarios was tracked by the app.

We found that most apps focused on passively delivering educational content. Providing education is important, as care partners report persistent unmet needs related to understanding ADRD as a disease process, including diagnosis, prognosis, and disease progression; long-term care and financial and legal planning; and management of cognitive and behavioral symptoms [4,5,26,27]. However, the extent of the effectiveness of passive learning content (eg, reading an article or watching a video) provided by these apps is unknown and may be limited as opposed to engaged active learning approaches that foster information retention [28-30]. In addition, care partners also reported the need for training, support for coordination across the caregiving network, connection to relevant resources, and social support [4,5,27,31-33]. Some apps did attempt to address care partners’ need for social support by offering forums, chats, and community features. However, we found that these features were often not functional or did not have active participation from users, limiting the app’s ability to fulfill their promise of social support. Furthermore, the apps were limited in functionality to support coordination across the caregiving network, with only 2 apps supporting coordination with other care partners and only 1 connecting care partners with clinicians. Overall, the limited functionality provided across most apps raises questions about their potential to improve care partner outcomes, as several recent systematic reviews and meta-analyses suggested that effective care partner interventions provide multiple components and social support [34-44].

Overall, the apps scored higher on functionality and aesthetics than on engagement and information quality. The apps, on average, scored just above minimally acceptable for functionality (mean 3.37, SD 0.99), which includes app performance, ease of use, navigation, and gestural design. Functionality is important for care partners because it reflects the potential of the app to meet basic care partner needs in terms of app usability. This score is a point lower than that indicated in the MARS rating reported in a 2020 study by Choi et al [22], which used the MARS to assess the quality of all ADRD-related apps, including those focused on care partners and those focused on the person living with ADRD. It is possible that the higher scores found by Choi et al [22] reflect a higher quality of apps designed for people living with ADRD, as a recent study by Guo et al [45] on rating mobile apps for people living with ADRD reported a similarly high functionality score.

On average, the apps scored as just above minimally acceptable for aesthetics (mean 3.24), including layout, graphics, and visual appeal. This is similar to the aesthetic scores reported by Choi et al [22] and lower than the average aesthetics score reported by Guo et al [45]. Aesthetics is an important dimension of quality that allows apps to stand out in the marketplace. Aesthetics can also facilitate emotionally positive experiences, which can improve user perceptions of the app [46,47].

However, on average, engagement, which included entertainment, customization, interactivity, and fit to the target group, was slightly below acceptable quality (mean 2.76). Similarly, the findings of both Choi et al [22] and Guo et al [45] reported that apps scored lowest on engagement, reporting just-below minimally acceptable quality and above minimally acceptable quality, respectively. These findings further confirm previous research that evaluated 8 commercially available apps for ADRD care partners and found that the majority provided mostly text-based information [48]. Below acceptable engagement scores are concerning, as engagement issues can lead to technology abandonment, reduced acceptance, or failure to use the app to its full potential [49,50]. For care partners, engagement may be critical, as they often experience high levels of demands associated with their caregiver role [31,51,52]. As demonstrated in other populations with chronic health conditions [53,54], engagement is important to sustain care partners’ attention when their attention is drawn to the many other demands they experience daily.

Information quality, which included information quantity, visual information, credibility, goals, and app description, also scored, on average, slightly lower than minimally acceptable (mean 2.95, SD 0.95). This is a point lower than the information quality score reported by Choi et al [22]. It is possible that this score difference could be because of information quality differences of the apps designed for people living with ADRD as their target users, which is further supported by a similar high score reported by Guo et al [45]. Information is a critical component to meeting care partners’ unmet needs, and low information quality may increase the likelihood of technology abandonment [55]. For example, recent research found that when care partners search for information and cannot meet the information need at that time, they often abandon the information behavior [18]. Furthermore, low-quality information is likely to reduce perceived usefulness, which has been shown to be a key factor influencing caregivers’ intention to adopt mobile health apps [56]. Lower scores on information are also concerning, as this score reflects that apps are often not tested for effectiveness in improving people living with ADRD or care partner outcomes, reducing the ability to safeguard against products that may not deliver on their advertised potential. Specifically, of the 17 apps, 7 (41%) had a mean information quality score that ranged from 1.17 to 2.50 and 11 had a mean subjective quality score that ranged from 1.00 to 2.50. The scoring of both dimensions indicates inadequate quality, which potentially heightens the risk of technology abandonment and loss of the intended impact for target users. Furthermore, apps often state goals without any way to measure or track goal attainment; therefore, there are no clear pathways provided to evaluate whether the stated goals are achievable.

Although most apps met the MARS requirement for minimal acceptability, it may not be sufficient to meet the needs of care partners of people living with dementia. Research on older adults’ technology acceptance indicates that they have a higher standard for technology acceptance [57,58]. As many care partners are older adults, raising the bar for acceptable mobile app quality may be critical to sustained care partner use. Furthermore, care partners experience high demands related to their caregiving role and managing complex symptoms and progressive decline and often experience suboptimal health outcomes such as high levels of burden, depression, and anxiety. Therefore, mobile apps may confer some level of risk and need to be held at a high standard so that they do not add burden or increase the risk of suboptimal health outcomes. In addition, an average score at the level of minimal acceptability may mask serious quality violations on one dimension that are counterbalanced by higher-than-average scores on other dimensions. For example, the above average–rated app Respite Mobile (mean MARS quality score 3.35) had a low information quality score (2.27) counterbalanced by particularly high scores on aesthetics (4.0) and functionality (4.25). Thus, minimum standards across dimensions may need to be imposed to avoid harm from counterbalanced weaknesses.

Overall, our ratings of the apps mirror some of those produced from a similar study by Choi et al [22], who also found app engagement scores to be lower than acceptable quality and further highlighted that their scoring differed based on the types of developers (ie, health care–related vs non–health care–related) and intended purpose (ie, awareness, assessment, and disease management). We lacked an appropriate sample to statistically compare differences between developer types. However, we similarly found that for overall mean scores, those developed by commercial entities were just below the minimally acceptable quality, whereas those developed by noncommercial entities were just above the minimally acceptable quality. This comparison further confirms our suggestion to establish higher standardized criteria for health information technology to meet the needs of the care partners of people living with dementia.

Considering the variability in app quality and the failure of many apps to attain acceptable overall and dimension-specific quality ratings, there is a need to adopt quality-focused design and development approaches. One such approach is UCD, introduced earlier and characterized by design driven by a foundational understanding of user needs, direct or indirect input from end users in the design process, and rigorous testing with representative samples of intended end users [16]. In participatory forms of UCD, sometimes called co-design, care partners can also actively contribute to design, leading to a higher likelihood that user needs and abilities are supported and accommodated [59]. UCD approaches can also be used to facilitate engagement through gamification and persuasive design. Furthermore, UCD-based emotional design can increase the quality of aesthetics and functionality [46,47].

### Limitations

The results of this study should be considered in light of certain limitations. Not all the raters in our study were experts in technology design. However, we had 3 expert raters who conducted training and acted as arbiters for inclusion decisions and MARS rating. In addition, as per the MARS approach, the raters were not users themselves. To enhance our understanding of the quality of mobile apps for care partners of people living with dementia, future studies should include user testing, such as usability testing and other user tests, alongside expert ratings. Furthermore, we did not rate apps that were available only to study participants. However, the apps we rated are currently available on the market to all users and not limited to the study inclusion and exclusion criteria and participation timelines. Related to this, we were able to rate only what we could access. This means that apps that malfunctioned during log-ins or were only available to customers of a specific health system were not reviewed.

We also identified the limitations of the MARS that should be considered. First, the MARS assumes a typical user and does not address diverse personas, such as users with diverse ages, physical and cognitive abilities, race, ethnicities, and urbanicity or rurality. Second, applying the MARS item definitions is somewhat subjective, and the definitions are not connected to norms, such as a database of prior MARS evaluations. We addressed this limitation through training by reconciling differences in the interpretation of definitions through discussions and consensus building. Third, the MARS does not include certain aspects of design that contribute to app quality, such as security, the design process used, data standards, and accessibility compliance.

### Conclusions

In evaluating the quality of publicly available apps for care partners of people living with ADRD, we found that apps, on average, are of minimally acceptable quality. Although we identified apps both above and below the minimally acceptable quality, many apps had broken features and were rated as below acceptable quality for engagement and information quality. Minimally acceptable quality is likely insufficient to meet the needs of care partners without potentially causing harm by increasing burden and stress. Future research should establish minimum quality standards across dimensions for mobile apps for care partners. The design features of high-quality apps identified in this study can provide the foundation for benchmarking these standards.

## Appendix

Multimedia Appendix 1 Agreement and disagreement rates of each dyad before the expert-based score reconciliation process.

## Abbreviations

ADRD: Alzheimer disease and related dementias
MARS: Mobile App Rating Scale
UCD: user-centered design
