# Supplementary material for: Quality of Mobile Apps for Care Partners of People With Alzheimer Disease and Related Dementias: Mobile App Rating Scale Evaluation
Source: JMIR Mhealth Uhealth. 2022 Mar 29;10(3):e33863. doi: 10.2196/33863 (PMC9006134; doi:10.2196/33863)
Supplement: Multimedia Appendix 1 [file mhealth_v10i3e33863_app1.docx]

**Appendix A.**

Agreement and disagreement rates of each dyad for exact same rating on a 1-5 scale prior to the expert-based score reconciliation process.

| Dyad | P/A | M/A | N/A | J/A | P/N | J/M |
| --- | --- | --- | --- | --- | --- | --- |
| Total | 95 | 19 | 76 | 57 | 19 | 57 |
| Agree | 56 | 8 | 31 | 17 | 9 | 22 |
| Disagree | 39 | 11 | 45 | 40 | 10 | 35 |
| % Agree | 59% | 42% | 41% | 30% | 47% | 39% |

Agreement and disagreement rates of each dyad rating within one point on a 1-5 scale prior to the expert-based score reconciliation process.

| Dyad | P/A | M/A | N/A | J/A | P/N | J/M |
| --- | --- | --- | --- | --- | --- | --- |
| Total | 95 | 19 | 76 | 57 | 19 | 57 |
| Agree | 88 | 17 | 68 | 39 | 15 | 44 |
| Disagree | 7 | 2 | 8 | 18 | 4 | 13 |
| % Agree | 93% | 89% | 89% | 68% | 79% | 77% |
